# A Three-Dimensional Reconstructive Study of Pelvic Cavity in the New Zealand Rabbit (*Oryctolagus cuniculus*)

**DOI:** 10.1155/2014/489854

**Published:** 2014-10-14

**Authors:** Sema Özkadif, Emrullah Eken, İbrahim Kalaycı

**Affiliations:** ^1^Department of Nursing, School of Health, Batman University, 72040 Batman, Turkey; ^2^Department of Anatomy, Faculty of Veterinary Medicine, Selçuk University, 42250 Konya, Turkey; ^3^Department of Geomatics Engineering, Necmettin Erbakan University Faculty of Engineering and Architecture, 42040 Konya, Turkey

## Abstract

The present study has been performed to reveal biometrical aspects and diameter-related differences in terms of sexes regarding pelvic cavity via three-dimensional (3D) reconstruction by using multidetector computed tomography (MDCT) images of pelvic cavity of the New Zealand rabbit. A total of 16 adult New Zealand rabbits, including 8 males and 8 females, were used in this study. Under anesthesia, the images obtained from MDCT were stacked and overlaid to reconstruct the 3D model of the pelvic cavity using 3D modeling software (Mimics 13.1). Measurements, such as the conjugate, transverse, and vertical diameters of the pelvic cavity, and the pelvic inclination were calculated and analyzed statistically. Biometrical differences of the pelvic diameters in New Zealand rabbits of both sexes were shown clearly. It was concluded that the pelvic diameters revealed by 3D modeling techniques can shed light on medical students who take both anatomy training and gynecological applications. The authors hope that the synchronization of medical approaches may give rise to novel diagnostic and therapeutic developments related to pelvic cavity.

## 1. Introduction

The impressive improvement in technology and convenience of cross-sectional data together with imaging methods provide an opportunity for the development of 3D models [1]. In rabbits the 3D anatomic structures of the paranasal sinuses have been revealed using their MDCT images and presented biometric properties of the sinuses and conchae [2].

Teaching and learning of complicated regions such as the pelvis become a problem because of limited standard education methods. Students slog up the 3D form of the pelvis due to the complex anatomy [1].

In human medicine, 3D digital modelshave been obtainedfrom 2D images of female pelvis and pelvis contents and strong teaching tools that will be used in anatomy education have been prepared [1]. 3D models having wide application areas were formed from high-resolution magnetic resonance (MR) images of pelvic organs [3].

The studies related with pelvis were performed not only on females but also on males. Some measurements of male pelvis were taken by 3D models obtained from computerized tomography (CT) images of male pelvis and their anatomic structures were presented [4]. Moreover, reconstruction of pelvis was performed and its cavity together with prostate volume was calculated. Therefore, an opportunity to get information about the difficulty level of the operation was found for the patient before the operation [5].

Sex discrimination from bone for adults is mostly performed on pelvis. 3D reconstruction was carried out by a computer program (Mimics 13.1) and obtained from 2D images of pelvis belonging to both males and females and the discrimination among sexes was presented with measured values [6].

3D CT images of pelvis were used in revealing anatomic properties and anatomy education as well as for determination and comparison of pathological situations in urology [7]. The studies about pelvis are not only limited inmedical field but also quite common in anthropological field. CT images and 3D modeling techniques also contribute to anthropological researches. Digital models obtained from CT provide an opportunity for removing and replacing a selected region. Thus, they help placing pelvis sections of the cadaver correctly [8].

It was observed that studies of veterinary medicine related with pelvis were benefited from CT images. The anatomic properties of Holstein-Friesian cows and Estonian Native Breed cows whose birth difficulties are commonly observed were presented by X-ray images and bones and the comparisons were made after measurement [9]. The external measurements of pelvis in Germen Holstein-Friesian cows were performed with caliper and its internal measurements were carried out with reconstruction images obtained from CT [10].

Biometric measurements of pelvis bones and diameters in dogs were performed in males and females of a crossbred race having dissection. The discrimination among sexes was revealed by measured values [11].

As it can be seen, 3D modeling of pelvis obtained by technological developments is used in anatomy education as well as medical diagnosis and treatment of diseases. In the literature there are not any studies related with 3D modeling of pelvic cavity in New Zealand rabbit and its biometric measurements. Just in one study, morphologies of bones forming back feet and os coxae of pelvis which belong to New Zealand rabbit and Plio-Pleistocene rabbit (*Hypolagus beremendensis*) were compared [12].

The objective of this study is to measure the certain pelvic diameters in New Zealand rabbits and reveal their biometric differences in terms of sexes, based on 3D reconstructions of pelvis-related MDCT images.

## 2. Material and Methods

The sacral region-related part of MDCT images was obtained from full screened body of New Zealand rabbits which was used in another project completed in early 2011 and supported by Coordinatorship of Selcuk University Scientific Research Projects with the Project number of 2009/056. The project mentioned above has already been approved by Ethic Board of Veterinary Faculty, University of Selcuk.

### 2.1. Age and Weight

In the study, 8 males and 8 females of a total of 16 New Zealand rabbits were used which were 1–1.5 years old and weighing between 3–3.5 kg.

### 2.2. Anesthesia

The rabbits were intravenously anesthetized with a mixture of 5 mg/kg ketamine-HCl (Ketamidor, RicherPharma AG, Austria) and 20 mg/kg propofol (Propofol amp., Fresenius Kabi, Austria).

### 2.3. MDCT Images

Under anesthesia, MDCT images of animals in prone position were obtained. The parameters of MDCT (Somatom Sensation 64; Siemens Medical Solutions, Germany) device were adjusted as follows: physical detector collimation, 32 × 0,6 mm; final section collimation, 64 × 0,6 mm; section thickness, 0,75 mm; gantry rotation time, 330 ms; kVp, 120; mA, 300; resolution, 512 × 512 pixel; and resolution range, 0,92 × 0,92. Dosage parameters and scanning were performed on the basis of standard protocols and literature [13, 14]. By this way, we tried to obtain radiometric resolution at the lowest radiation level and with optimum image quality (MONOCHROME2; 16 bit). High resolution MDCT images of pelvic cavity were obtained. After stocking obtained axial images as DICOM format, they were transferred to a personal computer loaded with 3-dimensional modeling program (Mimics 13.1 Meterialise Group, Belgium).

### 2.4. Three-Dimensional Reconstruction

**Figure 1:**
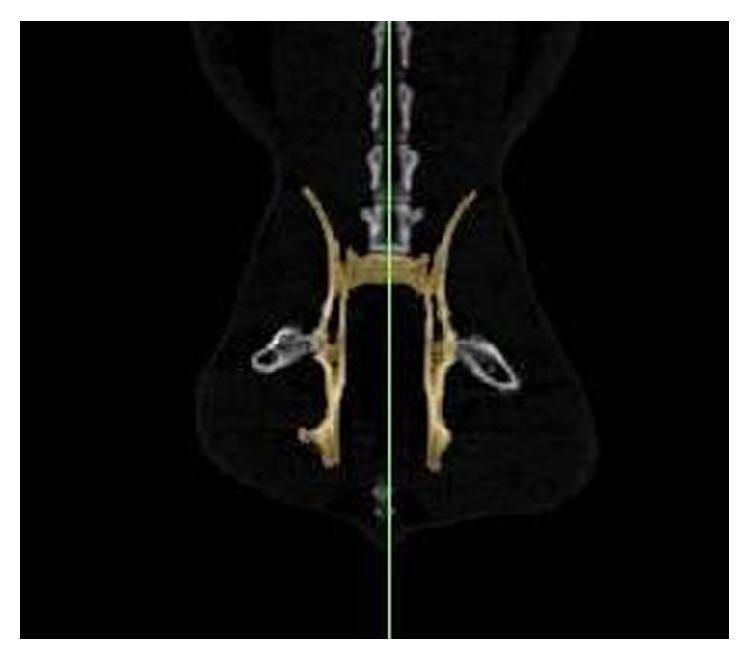
Colored designation of pelvis limits on coronal section.

At the first stage of automatic segmentation period, the limits of coxal, sacral, and coccygeal bones forming pelvic cavity were determined. In the places except the limits of the bones, the erasing process was applied section by section with the computer mouse and these places were cleaned (as shown in Figure 1). After controlling manual correction with naked eye and erasing unnecessary places, the images whose limits were determined were overlapped and then reconstruction was performed with 3D translator component of Mimics 13.1 program.

### 2.5. Measurements

**Figure 2:**
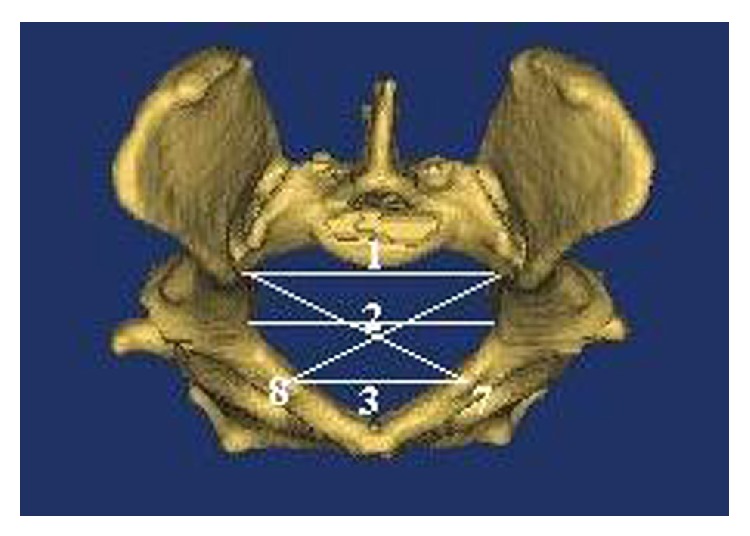
Measurements on facies cranialis of pelvic cavity.

**Figure 3:**
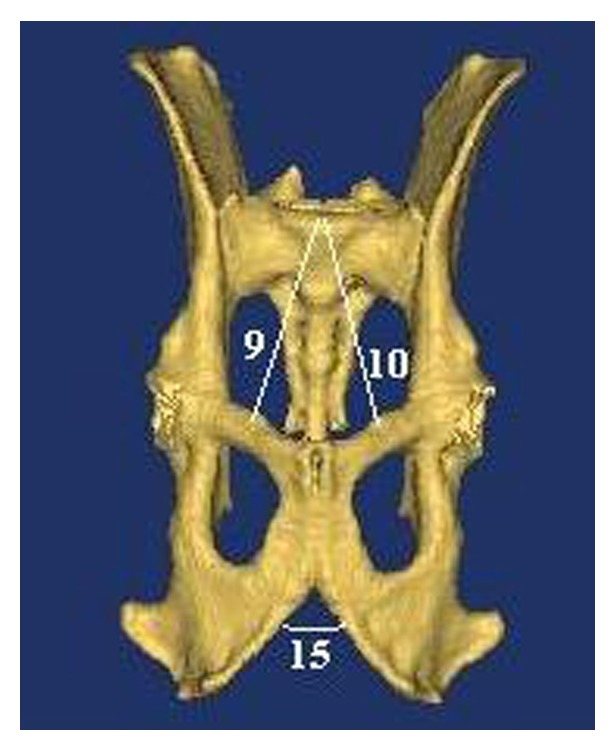
Measurements on facies ventralis of pelvic cavity.

**Figure 4:**
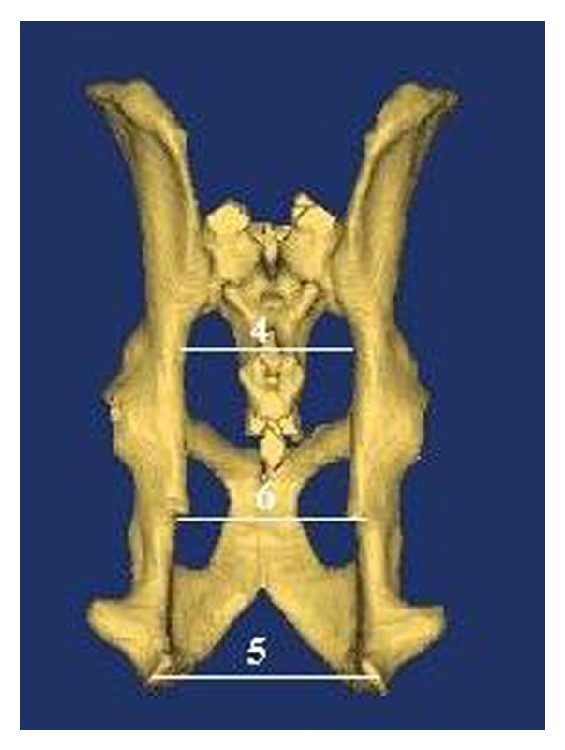
Measurements on facies dorsalis of pelvic cavity.

**Figure 5:**
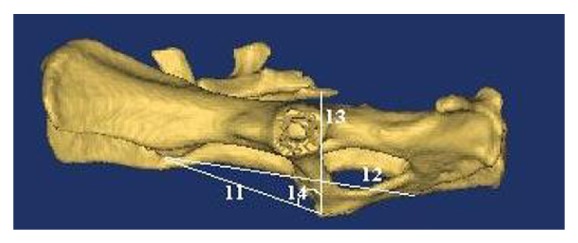
Measurements on facies lateralis sinistra of pelvic cavity.

In this study, on the basis of literature [15, 16], transverse diameters (dorsal transverse, intermediary transverse, ventral transverse, cranial transverse, caudal transverse, and medial transverse diameters), oblique diameters (right oblique diameter, left oblique diameter, right sacrocotyloid diameter, and left sacrocotyloid diameter), conjugate diameters (conjugata vera and conjugata diagonalis), vertical diameter, pelvic inclination, and angle between arcus ischiadicus were measured (as shown in Figures 2–5). After determining the limits of biometric measurements of pelvis, they were automatically calculated by the program.

*Nomina Anatomica Veterinaria* [17] was also used in terminology.

1. Dorsal transverse diameter: line/diameter linking the tips of the alae of the sacrum/ala ossis sacri (Figure 2).
2. Intermediary transverse diameter: line/diameter between the psoadic tubercles (the lesser psoas muscle tubercles). Note that tuberculum psoadicum (tuberculum m. psoas minoris) is a little crest that is of use for cleaving of m. psoas minor and is present approximately in the middle of linea arcuata (Figure 2).
3. Ventral transverse diameter: diameter between the iliopectineal eminences. Note that eminentia iliopectina is a sharp eminence present in the median nerve of os ilium and os ischii. Line arcuata ends at this eminence (Figure 2).
4. Cranial transverse diameter: line/diameter linking the greater/major sciatic notch (Figure 4).
5. Bituberous (caudal) transverse diameter: line/diameter between ischial tuberosities (Figure 4).
6. Bispinous (medial) transverse diameter: line/diameter between ischial spines (Figure 4).
7. Right oblique diameter: line/diameter from the right sacroiliac joint to the left iliopectineal eminence (Figure 2).
8. Left oblique diameter: line/diameter from the left sacroiliac joint to the right iliopectineal eminence (Figure 2).
9. Right sacrocotyloid diameter: line/diameter from the promontory of the sacrum to the right iliopectineal eminence (Figure 3).
10. Left sacrocotyloid diameter: diameter from the promontory of the sacrum to the left iliopectineal eminence (Figure 3).
11. Conjugata vera: line/diameter drawn from the tip of the sacral promontory to the cranial end of the symphysis pelvina/pubis (Figure 5).
12. Conjugata diagonalis: line/diameter drawn from the tip of the sacral promontory to the caudal end of the symphysis pubis/pelvina (Figure 5).
13. Vertical diameter: the vertical diameter/line extends from the cranial end of the symphysis pubis (pelvina) to the ventral surface of the sacrum (Figure 5).
14. Inclinatio pelvis: the inclinatio pelvis is the angle between the conjugate and vertical diameters (Figure 5).
15. The angle between arcus ischiadicus (Figure 3).

### 2.6. Statistical Analysis

Statistical analysis was carried out with SPSS 15.0 windows computer packaged software. Independent samples* t*-test were performed and mean and standard deviation values of biometric measurements which belong to the pelvic cavity of male and female New Zealand rabbits were given. Statistical significance was recorded as *P* < 0.05.

## 3. Results

Statistical analysis of biometric measurements was performed by presenting 3D modeling of pelvic cavity; the 3D reconstruction was also carried out. According to statistical results, the differences between measured values of pelvis diameters in terms of sexes were determined at the level of *P* < 0.05.

When Table 1 is investigated, a difference (*P* < 0.05) was determined between dorsal transverse, cranial transverse, caudal transverse, medial transverse, right oblique, and left oblique diameters, conjugata vera, conjugata diagonalis, vertical diameter, and inclinatio pelvis and the angle between arcus ischiadicus from measurement values of pelvic cavity belongs to male and female rabbits. There is no significance between male and female New Zealand rabbits in terms of intermediary transverse, ventral transverse, right sacrocotyloid, and left sacrocotyloid diameter values.

In Table 2, it was determined that there is no statistical difference between right and left measurements of oblique diameterand sacrocotyloid diameter for male and female rabbits.

## 4. Discussion

According to biometric measurements of pelvic cavity diameters belongs to males and females, there was statistical difference between dorsal transverse, cranial transverse, caudal transverse and medial transverse diameters among transverse diameters of pelvis, between right oblique and left oblique diameters among oblique diameters of pelvis, between conjugata vera and conjugata diagonalis among conjugate diameters of pelvis and between vertical diameter; inclinatio pelvis and the angle between arcus ischiadicus and these values were higher for females than males. The information related with the distance between symmetrical parts of pelvis is always higher in females and the angle between arcus ischiadicus is wider in females according to the information present in the literature [15]. This situation, that is, the size of female pelvis is bigger than that of male pelvis, is also similar for human beings [18].

Right and left oblique diameters of female pelvis in human beings are equal to each other [16]. In New Zealand rabbits, mean value for right oblique diameter of male is 25.13 mm and mean value for left oblique diameter of male is 25.80 mm; mean value for right oblique diameter of female is 29.05 mm and mean value for left oblique diameter of female is 27.98 mm. Finding no statistical difference between right and left oblique diameters in both male rabbits and female rabbits is also in accordance with the situation of human beings. Moreover, there is no difference between right and left sacrocotyloid diameters of females in human beings [16]. This situation also resembles the New Zealand rabbits. In other words, while right sacrocotyloid diameter of male mean value is 26.43 mm and mean value for left sacrocotyloid diameter of male is 26.75 mm, mean value for right sacrocotyloid diameter of female is 27.59 mm and mean value for left sacrocotyloid diameter of female is 27.23 mm. These values have no statistical difference in terms of left and right sides.

When measurement values of cranial and caudal pelvic cavities having importance during birth were considered, the mean values of dorsal transverse diameter (22.5 mm), right oblique diameter (29.05 mm), left oblique diameter (27.98 mm), and vertical diameter (22.48 mm) which belong to cranial pelvic cavity of females in New Zealand rabbits were higher than the mean values of dorsal transverse diameter (20.95 mm), right oblique diameter (24.69 mm), left oblique diameter (25.80 mm), and vertical diameter (20.34 mm) of males and were statistically significant. In the morphological research of Nahkur et al. [9] related with Estonian Holstein Breed and Estonian Native Breed race cows, it was determined that intermediary transverse diameter and vertical diameter of Estonian Holstein Breed had considerably high values and there was a strong statistical correlation in the formation of cranial pelvic cavity field. It was also indicated that dorsal transverse diameter was not so effective on the shaping of upper edge of cavity specified by intermediary transverse diameter. In our study, it was determined that there was a statistical difference between males and females in terms of dorsal transverse diameter of rabbits and it was effective on the formation of cranial pelvic cavity. While ventral transverse diameter had little effect on Estonian Holstein Breed race cows [9], it had no statistical importance for New Zealand rabbits.

There were not any statistical differences between males and females in ventral and intermediary transverse diameter, oblique diameter, and conjugate vera in dogs [11]. It was also the same in New Zealand rabbits in ventral and intermediary transverse diameter as in dogs. But contrary to the dogs, there were statistical differences in oblique diameter and conjugate vera in New Zealand rabbits between males and females in our study.

The angle of arcus ischiadicus being bigger (*P* < 0.05) in female New Zealand rabbits than males indicated that pelvis took a more flat and wide shape and the angle being smaller (*P* < 0.05) in male rabbits than females indicated that pelvis had a narrower structure. In other words, as the angle of arcus ischiadicus increases, the width of pelvis also increases.

In anatomic studies, there are many advantages of 3D reconstructive researches performed with 3D programs from CT images. It is very important in terms of ethics that CT images of animals were taken under general anesthesia without killing them and they survived after waking up. Since 3D models obtained from CT images can be turned whichever direction we want, it enables understanding of anatomic parts completely and taking desired measurement values. Together with using high technology and computer programs, the numbers of qualified and modern anatomic studies are also increasing. The accuracy of biometric measurement values obtained in anatomic studies has been proved. As interslice distance of CT images decreases, the reliability coefficient of 3D biometric values obtained from organ or tissues also increases [19]. It is also a fact that CT-related 3D reconstructive anatomic studies have some limitations. Since technological devices are quite expensive, absence of tomography in many veterinary faculties or private veterinary hospitals makes planning of CT studies on animals difficult or decreases the possibility of using the results obtained from these studies in clinics. Moreover, an expert staff should be employed in clinics to evaluate the results of tomography. While getting permission to take CT images of animals in medical faculties or private hospitals, unwillingness and/or disapproval of radiography experts are important handicaps.

Consequently, on basis of the pelvic cavity-related data obtained from 3D reconstructions of MDCT images in New Zealand rabbits, the biometric differences of the pelvic diameters between genders were revealed clearly. It has been proposed that both biometric perspectives of pelvic cavity and the 3D reconstruction technique performed in this work add a new point of view to the future studies on reconstructive studies. The authors also suggest that this study using high technology may add a modern dimension to anatomical education and contribute considerably to the present anatomical knowledge related to rabbit pelvis.

## Figures and Tables

**Table 1:** Statistical results of biometric measurements belonging to pelvic cavity obtained as a result of 3D reconstruction (mm ± SD).

	Male (*n* = 8)	Female (*n* = 8)
Dorsal transverse diameter∗	20.95 ± 0.38	22.50 ± 0.92
Intermediary transverse diameter	23.25 ± 0.34	23.23 ± 0.98
Ventral transverse diameter	14.54 ± 0.32	14.55 ± 0.27
Cranial transverse diameter∗	22.11 ± 0.05	22.44 ± 0.31
Bituberous (caudal) transverse diameter∗	23.56 ± 0.17	26.76 ± 1.10
Bispinous (medial) transverse diameter∗	21.97 ± 0.74	25.30 ± 1.36
Right oblique diameter∗	25.13 ± 0.44	29.05 ± 1.42
Left oblique diameter∗	25.80 ± 0.39	27.98 ± 0.64
Right sacrocotyloid diameter	26.43 ± 1.37	27.59 ± 1.43
Left sacrocotyloid diameter	26.75 ± 0.62	27.23 ± 0.56
Conjugata vera∗	27.98 ± 0.56	29.94 ± 0.82
Conjugata diagonalis∗	43.24 ±1.20	47.13 ± 3.23
Vertical diameter∗	20.34 ± 0.39	22.48 ± 0.19
Inclinatio pelvis∗	72.44 ± 1.30	76.01 ± 2.00
The angle between arcus ischiadicus∗	66.32 ± 0.83	75.18 ± 1.11

^*^ *P* < 0.05. Data expressed as the mean ± SD.

**Table 2:** Statistical comparison of biometric measurements belonging to left and right sides of pelvic cavity obtained as a result of 3D reconstruction (mm ± SD).

		Right (*n* = 8)	Left (*n* = 8)
Male	Oblique diameter	25.13 ± 0.44	25.80 ± 0.39
	Sacrocotyloid diameter	26.43 ± 1.37	26.75 ± 0.62

Female	Oblique diameter	29.05 ± 1.42	27.98 ± 0.64
	Sacrocotyloid diameter	27.59 ± 1.43	27.23 ± 0.56
